# Polymer Microtip on a Multimode Optical Fiber as a Threshold Volatile Organic Compounds Sensor

**DOI:** 10.3390/s22031246

**Published:** 2022-02-07

**Authors:** Paweł Marć, Monika Żuchowska, Iwona Jakubowska, Leszek R. Jaroszewicz

**Affiliations:** Faculty of Advanced Technologies and Chemistry, Military University of Technology, 2 Gen. S. Kaliskiego St., 00-908 Warsaw, Poland; monika.zuchowska@wat.edu.pl (M.Ż.); iwona.jakubowska@wat.edu.pl (I.J.); leszek.jaroszewicz@wat.edu.pl (L.R.J.)

**Keywords:** photopolymerization, optical fiber refractive index sensors, vapor detection

## Abstract

Polymer microtips are 3D microstructures manufactured on the end face of an optical fiber by using the photopolymerization process. Such micro-optic elements made on a multi-mode optical fiber were previously tested as a transducer of refractive index sensor. These studies were an inspiration to investigate the possibility of using this type of transducer to measure the presence of volatile organic compounds in the air. The experimental results of microtips polymerized with UV and VIS were reported. It was possible to detect the presence of five different volatile compounds in the air due to the sensitivity of the transducer to the refractive indices changes. These changes were induced by the vapors condensed on the microtip surface. The measured time responses have shown that the return loss decreases rapidly as the microtip is inserted inside a glass vial filled with the tested compound. Moreover, correlations between calculated dynamic ranges and refractive indices and volumes of the volatile compounds inside the vials were negligible. Therefore, this type of sensor can be categorized as a condensed material threshold sensor. This sensor can be used in warning systems for monitoring leakages of pipelines carrying volatile chemicals.

## 1. Introduction

In recent years, optical fiber sensor technology has attracted considerable interest. This is due to its many unique properties, such as high sensitivity, fast response, compact size immunity to electromagnetic interference, and corrosion resistance [1]. Most of these advantages allow this technology to be used in various research fields. Sensors based on long-period gratings [2,3,4], Bragg gratings [5,6,7], tapered fiber [8,9], and interferometers [10,11] are commonly used. However, this technology is still evolving, new systems and configurations are being implemented.

Particular interest in using optical fiber as a transducer has led to the development of a lab-on-fiber technology as a new class of sensing platforms [12,13]. In this class, lab-on-tip platforms include functional materials integrated at the optical fiber end face [13]. Functional materials as a combination of metal and polymer, with the size of micro- or nanostructures manufactured by means of a UV-imprint transfer, were used to design the proper sensor transducer [14]. It is also possible to transform this part of the optical fiber into a glass microtip aiming to improve the coupling between the single-mode optical fiber (SMF) and lasers or laser diodes [15,16,17,18,19], or scanning near-field optical microscopy (SNOM) [20]. These elliptic-cone-/wedge-shaped-/conical-wedge-shaped end faces are fabricated by using different processes such as grinding [15], polishing [16], micromachining of a pulsed CO_2_ laser [17,20], grinding and polishing with heating in a fusing splicer [18], and chemical etching [19,20]. Another concept of the microtip fabrication is the use of the photopolymerization process. Originally, this process was used to make a protective coating on an optical fiber. First, a thin film of a liquid monomer mixture is applied to the side surface of the optical fiber, and then it is quickly cured with UV light when the optical fiber is drawn. The reliability of this process made it the only one acceptable and used so far [21]. However, the photopolymerization process was implemented to make a 3D polymer micro-optical element (microtip) on the end face of the fiber as an extension of its core [22,23,24,25,26,27]. Previous studies have shown the possibility to fabricate the microtips on various optical fibers (i.e., SMFs [22,23,25,26], photonic crystal [24] and multi-mode fiber (MMF) made with silica [27] and plastic [25]). The most important applications of such micro-optical element are in scanning optical microscopes (SOM) [28], optical fibers interconnections [29,30,31], optical fiber couplers/splitters [32], VCESEL illumination profile controllers [33], and sensors [34,35,36]. In the first application, the SMF was applied to achieve a submicron resolution of the designed optical fiber-based SOM [28]. Low loss connections between a pair of SMFs [29,31] and silica and plastic MMFs [30] were also reported. The extended idea of a polymer connector was used to manufacture a 2 × 2 micro-bridge between four standard telecommunication SMFs described as an optical fiber coupler/splitter [32]. Furthermore, polymer microtips were applied to enhance an outgoing light beam profile of the VCESELs [33]. Moreover, a microtip produced on the silica MMF as a transducer of the refractive index (RI) sensor was described [33,34,35]. The concept of using such a microelement as a RI sensors transducer was validated by the measurement of return loss of optical backscattered signal while the microtip was immersed in various reference liquids. The highest sensitivity of 208 dB/RIU, with a dynamic range of 32 dB, for the RI range within 1.35–1.48 was achieved [36]. These studies have suggested new potential applications for polymer microtips.

The possibility of detecting volatile organic compounds (VOCs) by using optical fiber RI sensors has been investigated [37,38,39]. Transducers in these sensors were different. In the first design, the MMF end face was coated with gold nanoparticles functionalized with a metal–organic framework [37]. Measurement of the wavelength shift of the absorbance peak of localized surface plasmon resonance (LSPR) allowed one to identify the presence of the selected VOC. Another concept was based on coating an optical fiber Bragg grating (FBG) with a poly(dimethylsiloxane) (PDMS). The PDMS layer exposed to VOC swelled, which generated a tensile force on the FBG and a Bragg wavelength shift was observed [38]. In the next design, a mesoporous film based on nanoparticles along with an organic moiety of poly(allylamine hydrochloride) polycation infused with a functional compound calixarene to modify the sensing layer of the long period grating (LPG) was used. This allowed one to measure RI change as a shift of the absorption peak induced by the selected VOC [39].

In this paper, the detection of the selected VOCs presence in the air by using a polymer microtip manufactured on the end face of MMF was presented. The microtip is a transducer of this sensor and uses the phenomenon of attraction and repulsion in terms of van der Waals forces of the tested VOC. Attracted VOC particles cover the surface of the microtip and form liquid droplets as the result of condensation which changes the RI at the interface between the microtip and the external material. This change can be measured by an optical backscatter reflectometer [36]. To test the proposed sensor, the following volatile materials were used: trimethyl phosphate (TMP), 1,4-thioxane (THX), acetone, toluene, and ammonia aqueous. The last three compounds were selected due to their RIs which are comparable with TMP and THX, but their vapor pressures are different. Therefore, the possible selectivity of the polymer micro-transducer was evaluated.

Preparation of the optimal microtip was preceded by several tests which allowed us to determine the correct chemical composition of the monomer mixture. Mixtures which could be polymerized with UV or VIS light, depending on chemical components, were used. Finally, two mixtures were selected and used in all further presented experiments. One consisting of a monomer and an initiator was dedicated to the UV polymerization, and the other needed an additional co-initiator and was used for the VIS polymerization. Shaping the geometry of such microelements was possible thanks to using a proper type of optical fiber, monomer mixture, and tuning technological process parameters [27]. Microtips produced in this way as a RI sensitive optrode was used. The sensor was tested in a reflection configuration. However, the analysis of its transmission properties will be the subject of further research.

## 2. Microtip Manufacturing and Sensor Preparation

**Figure 1:**
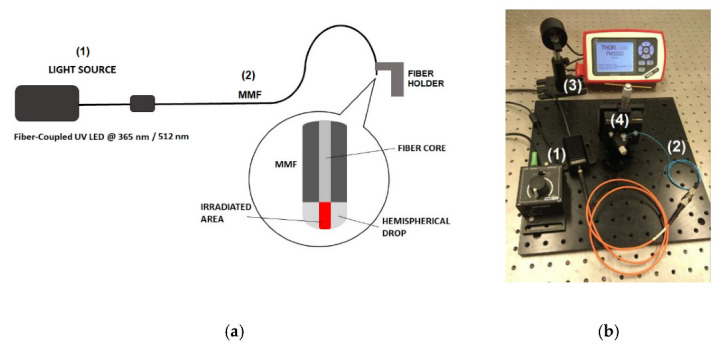
Sketch (**a**) and image (**b**) of the experimental set-up for the microtip manufacturing: (1) light source (UV LED-M365FP1 or VIS LED-M530F2 with LEDD1B driver, both *ThorLabs*, Newton, NJ, USA), (2) MMF 105, (3) power meter with sensor (S140C with PM100D console, both *ThorLabs*, Newton, NJ, USA), (4) fiber holder.

Manufacturing of an optical fiber microtip based on the photopolymerization process was previously described many times [22,23,24,25,26,27,28,29,30,33,34,35,36]. In Figure 1, the sketch (a) and the image (b) of the manufacturing set-up were presented. In all presented experiments, the step-index silica glass MMF FG105LCA (Thorlabs, Newton, NJ, USA) was applied. Its main parameters are as follows: core diameter of 105 µm, clad-ding diameter of 125 µm, numerical aperture equal to 0.22. In this paper, it is called MMF 105.

Briefly, the MMF 105 (2) was coupled to the UV LED or VIS LED (1) (UV-M365FP1 or VIS-M530F2 with LEDD1B driver, both *ThorLabs*, Newton, NJ, USA). At the first stage of experiment, the other cleaved end of MMF 105 was connected to the optical power meter (3) (S140C with PM100D console, both *ThorLabs*, Newton, NJ, USA) to set proper value of this parameter for photopolymerization. Then, this end of the fiber was placed in the fiber holder (4) and a monomer mixture drop was deposited. Illumination of a hemispherical monomer drop placed horizontally on the optical fiber end face formed a microtip as an extension of the core (inset in Figure 1a). After removing the unpolymerized material with isopropanol, the microtip was ready to be use as a sensor head for further testing.

**Figure 2:**
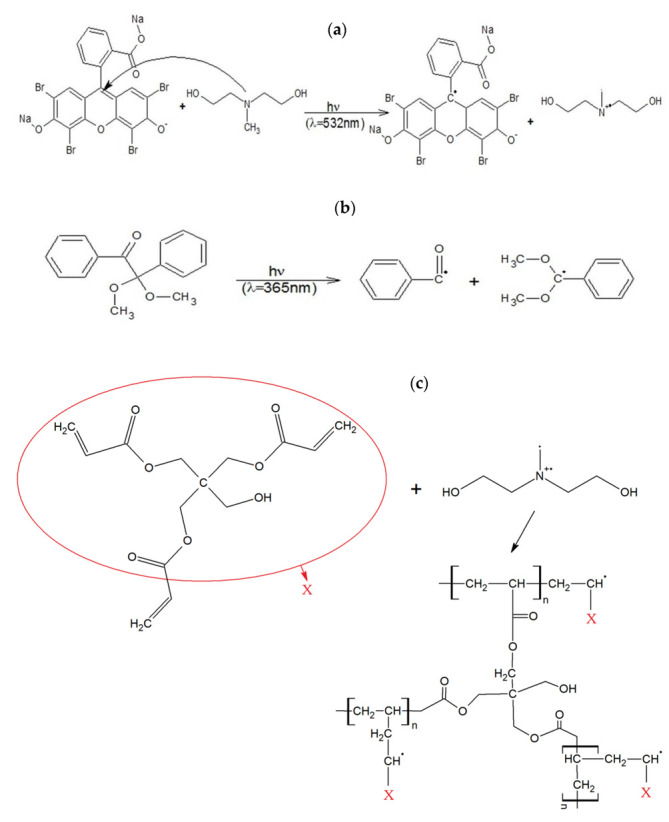
Examples of a photopolymerization reactions courses: (**a**) initiation excited by the VIS photon with a wavelength of 532 nm, (**b**) initiation excited by the UV photon with a wavelength of 365 nm, (**c**) PETA mixture—propagation and termination shown as an example for one of the C=C bonds in this 3-functional monomer (X—further polymerization).

In the experiments, two types of monomer mixtures based on a 3-functional monomer pentaerythritol triacrylate (PETA; *Sigma-Aldrich,* Saint Louis, MO, USA) were prepared. 2,2-Dimethoxy-2-phenylacetophenone (DMPAP; *Sigma-Aldrich,* Saint Louis, MO, USA) as a photo-initiator to make it UV sensitive was used in the first mixture and in the latter Eosin Y disodium salt (*Sigma-Aldrich,* Saint Louis, MO, USA) was used due to its VIS light sensitivity. Additionally, for the VIS mixture, methyl diethanolamine (MDEA; *Sigma-Aldrich,* Saint Louis, MO, USA) as a co-initiator was applied. All above-mentioned chemical compounds were used without any further purification. Figure 2 shows the possible courses of the photoinitiation and polymerization reactions. Figure 2a,b show the initiation process. The former one, a radical cation formation on tertiary nitrogen is excited by the VIS photon (a photon with a wavelength of 532 nm was shown as an example) in the presence of eosin and MDEA. The latter reaction is the UV-induced decomposition of DMPAP into an active (i.e., initiating) element of the benzoyl radical and a passive element of the acetal radical (as an example, photon with a wavelength of 365 nm was shown). Moreover, in Figure 2c, one of the possible courses of the polymerization reaction of PETA was presented. This stage of radical polymerization is chain growth, known as propagation. This increase consists in the successive attachment of the monomer molecules, initially to the free radical formed in the polymerization initiation stage, and then to the still growing macroradia.

The free radical uses one electron from the pi bond to form a more stable bond with a carbon atom. The second electron goes back to the second carbon atom, turning the entire molecule into another radical. This starts the polymer chain. The last stage ends the chain. Termination may occur through several different mechanisms.

Connection of two active chains ends can occur as a simply joining and/or their disproportionation reaction. Radical disproportionation occurs when the hydrogen at one end of the chain is separated from the other to form a polymer with a saturated or unsaturated terminal group. In addition to the reaction shown below, polycondensation, or even a mixture/combination of these reactions, is also possible due to the three active sites (C=C bond) in this monomer.

**Figure 3:**
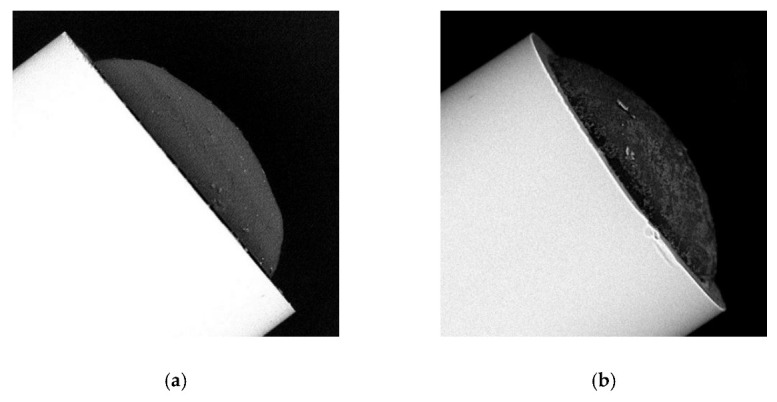
SEM image of polymer microtip on MMF 105, P = 30 µW, T = 60 s, produced using (**a**) UV LED (UV microtip); (**b**) VIS LED (VIS microtip).

The most important parameters of light sources used in the photopolymerization process were: UV LED (the central wavelength of 365 nm and FWHM around 5 nm) and VIS LED (the central wavelength of 512 nm and FWHM around 20 nm) [27]. Spectral characteristics of these sources were adapted to the properties of the prepared (as described above) two monomer mixtures. Therefore, in the next paragraphs, microtips have been divided into two categories: those made with the use of the UV LED (UV microtip) or those made with the VIS LED (VIS microtip). Moreover, taking into account reflective properties of microtips, the two most important process parameters (i.e., optical power (P) and exposure time (T) were optimized). Their optimal values were of 30 μW and 60 s, respectively [27]. Geometry characterization of UV microtips and VIS microtips produced with optimal parameters at the end face of MMF 105 were carried out based on the scanning electron microscope (SEM) images. Examples of the produced microtips were shown in Figure 3. Both presented microtips have similar geometry and hemispherical shape. Surfaces are smooth as it was expected using LED sources for photopolymerization in the experiment [27,34,35,36]. Geometry of the presented UV microtip [Figure 3a] is as follows: a base diameter of 96 ± 1 μm and a high one of 24 ± 1 μm. Furthermore, VIS microtip [Figure 3b] has a base diameter of 104 ± 1 μm and a high one of 26 ± 1 μm. Measurements were carried out based on built-in software of the used SEM (Phenom Pro, *FEI (Thermo Fisher Scientific)*, Hillsboro, OR, USA).

## 3. Experiment

**Figure 4:**
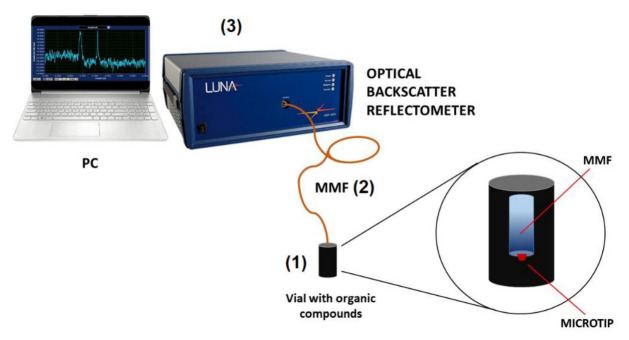
Measurement set-up of the VOC sensor: (1) glass vial with VOC, (2) MMF 105, and (3) OBR with PC.

The experimental set-up presented in Figure 4 consists of glass vial with VOC (1), MMF with microtip (2), Optical Backscatter Reflectometer (3) (OBR 4600, *Luna Technologies*, Roanoke, VA, USA), and PC. MMF 105 was coupled to the OBR and as a measured value in this experiment was used the return loss of the backscattered signal. In this system, the return loss is calculated as the ratio of time-integrated amplitudes of selected pulses to the amplitude of pulses in the whole period. A detailed description and principles of operation of this device, adapted from OBR 4600 user guide, are presented in [27].

MMF 105 with a manufactured microtip was placed inside a steel needle and then was pierced through the septum of the vial screw cap. A selected amount of VOC was deposited inside the vial and the evaporation process started immediately. VOC vapors are attracted to the surface of the microtip, and the condensation process takes place when the VOC concentration is sufficient. The environment around the microtip changes from gaseous (air) only to partially liquid due to the droplets’ formation [40]. This results in the average RI change around the microtip and the measured return loss level. This process allows one to characterize optical properties of the manufactured microtip as the VOC sensor transducer. Based on the rate of return loss change, the dynamics of the proposed sensor were determined.

The experiments began with wider trimethyl phosphate (TMP) tests, followed by an evaluation of the remaining four compounds. Vials with a volume of 5.02 mL were filled with 50 µL and 100 µL of the liquid TMP. For these two volumes, only a part of the liquid TMP transit into the gas phase (vapor pressure of 0.85 mm Hg at 25 °C). Preliminary tests have shown that thermodynamic equilibrium between gas and liquid phase is reached of around one hour. For a shorter time, insufficient liquid TMP was able to evaporate, and the concentration can vary in both time and in volume. In turn, leaving the TMP in the vial for longer than the optimal time causes that heavy TMP vapors to settle back to the bottom of the vessel, so the TMP concentration in the remaining volume is too low and might be nonuniform. Moreover, it was observed that evaporation time depends on the amount of liquid TMP poured into the vial, also. The maximum concentration of TMP in the headspace was determined experimentally and was around 0.28 mg/mL.

## 4. Results

**Figure 5:**
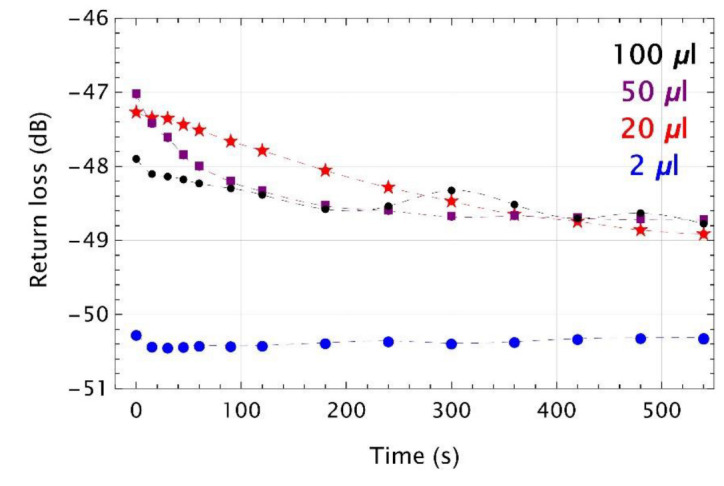
Time responses as return loss vs. time for MMF 105 without microtip with the presence of TMP in the glass vial of the volumes of: 2 µL (blue), 20 µL (red), 50 µL (purple), and 100 µL (black). Colored markers are experimental points, while the dashed lines are their cubic polynomial interpolations.

In the first part of the experiment, as a reference, the MMF 105 without a microtip was exanimated. It was successively placed inside the vials filled with different volumes of TMP and after each measurement it was cleaned and dried. Examples of time responses plotted as return loss vs. time were shown in Figure 5**.** For these experiments, glass vials with TMP volumes of 2 µL, 20 µL, 50 µL, and 100 µL were used.

For cases where TMP volume was of 20 µL and above, the return loss slightly decreases with time. For a 20 μL volume, these changes are linear to of around 250 s and next decreased nonlinearly. For 50 μL, changes were nonlinear over the entire range and saturation level of around −48.5 dB after 300 s is reached. An increase of the TMP vapors concentration inside the vial causes more TMP molecules to be deposited at the end face of MMF 105. When the condensation process occurs, a thin liquid film on the glass surface can be formed and the RI at the interface between the glass and the liquid TMP changes. Dynamic range of return loss is not significant and reaches of around 2 dB for 20 µL and 50 µL, and of around 1 dB for 100 µL. For the last data set, fluctuations of return loss are observed, which are probably caused by evaporation and condensation process. Time response for a 2 μL volume of TMP indicates that for this amount of VOC such type of transducer is out of detection level. Dynamic ranges estimated based on the above data were compared with previous results, which was of around 20 dB [37]; the return loss value was −47.1 dB in the air and −66.8 dB in the reference liquid with the RI equal to 1.4. Deposition of the liquid TMP layer, the RI of which is of 1.3967 [41], at the end of the optical fiber did not give dynamics at this level. The amount of the vapors attracted to the surface of the optical fiber end face is substantially smaller to induce an effect comparable to that of a liquid.

**Figure 6:**
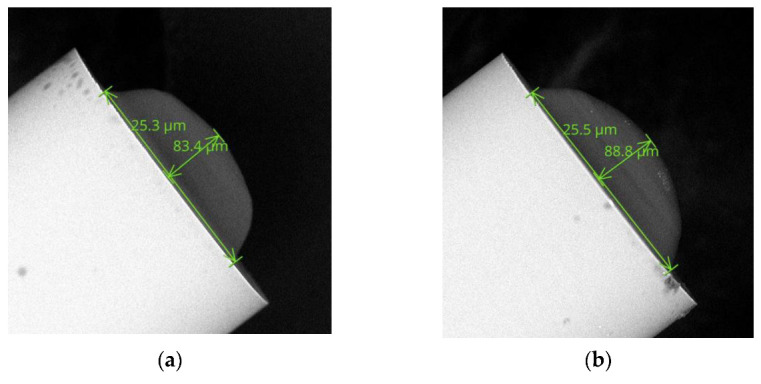
SEM images of UV microtips with the following base diameters and heights: (**a**) 83.4 μm, 25.3 μm; (**b**) 88.8 μm, 25.5 μm.

In the next step of experiments, the MMF 105 with the optimal microtip was tested. In Figure 6, examples of SEM images of UV microtips were shown. Both microelements have hemispherical shapes and similar dimensions (i.e., base diameters: 83.4 μm and 88.8 μm and heights: 25.3 μm and 25.5 μm). These data confirm the acceptable repeatability of the manufacturing process.

**Figure 7:**
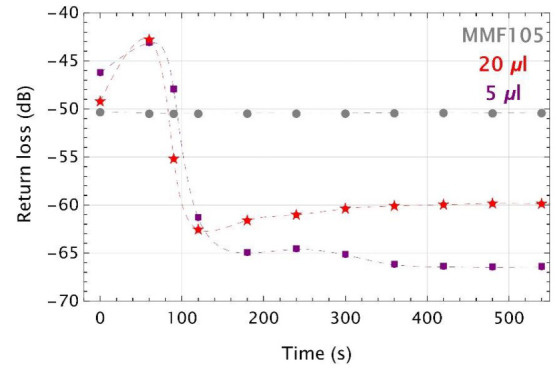
Time responses of MMF 105 with UV microtips with the presence of TMP in the glass vial of the volumes of: 5 µL (purple), 20 µL (red). Points joined with the gray dashed line are data of MMF105 without microtip tested for a TMP volume of 2 µL. Colored markers are experimental points, while the dashed lines are their cubic polynomial interpolations.

Based on previous research [37], it was determined that the UV microtips shape and size presented in Figure 6 are optimal. Two plots of experimental time responses of such transducers exposed on the tested VOC were shown in Figure 7. Colored markers are the measurement points while dashed lines are their cubic interpolations for TMP volumes of 5 µL (purple) and 20 µL (red). Additionally, experimental data for MMF 105 without the microtip are shown as gray dashed lines when it was exposed to the vapors of a volume of 5 µL TMP.

For the MMF 105 with microtips, the return loss decreases rapidly within the range of 60 s–120 s. Above 120 s, both curves are approximately flat with small fluctuations of the measured signal. In both cases, dynamic ranges were of around 20 dB and these changes show that this kind of sensors is a threshold one.

**Figure 8:**
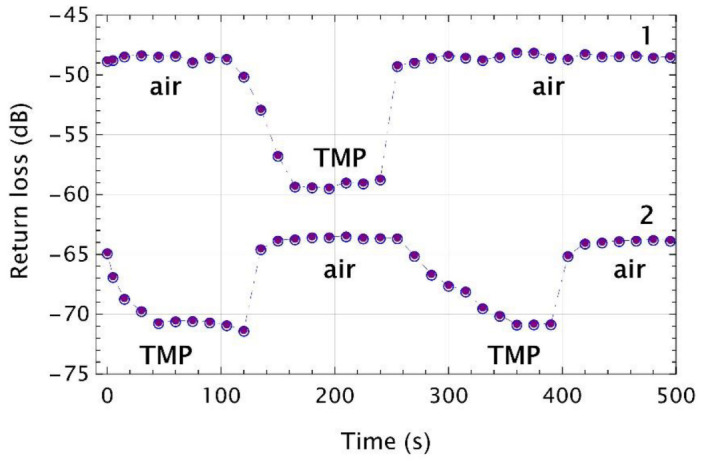
Time responses of the MMF 105 with a VIS microtip when the microtip was sequentially inserted into and removed from the glass vial filled with a 50 µL TMP. Empty circles with dots are experimental points, while the dashed lines are data interpolation.

Further studies were focused on the evaluation of the measurements’ repeatability of the sensor with VIS and UV microtips. In Figure 8, the experimental data of two VIS microtips exposed to 50 μL of TMP were shown.

Data set 1 represents a transducer which was measured first in the air up to 100 s and the average return loss level was of about −48.6 dB. Then, the microtip was placed in the vial with TMP vapors and after 40 s return loss decreased of around 10.6 dB to −59.1 dB. After next 100 s, the transducer was again taken out from the vial and return loss was back to the previous level of around −48.5 dB. For dataset 2, the transducer was put into the vial immediately after starting the measurement. Due to the initial return losses of −64.9 dB, a decrease of only 5.9 dB was observed to minima of around −70.6 dB which was reached after 75 s, when it was exposed to TMP vapors. After next 75 s, it was taken out of the vial and measured in the air for next 150 s. The average level of return loss in the air was of around −63.8 dB. Then, the microtip was put into the vial for the second time for 150 s and the saturation level was of −70.6 dB. Finally, it was again measured in the air for which return loss returned to its previous level to −63.8 dB.

**Figure 9:**
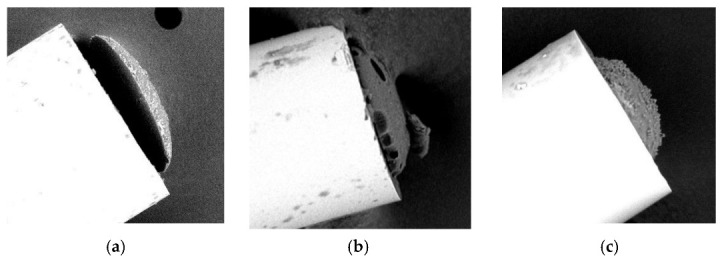
SEM images illustrating technological issues of the microtips manufacturing process: (**a**) partial delamination, (**b**) gas bubbles, and (**c**) compounds aging.

Both microtips used in the above experiment were made with the same process parameters but return loss levels were different. Such experiments were carried out with UV microtips, as well. Therefore, a critical evaluation of the manufacturing process was conducted. It allows to identify three main problems which significantly reduce back reflection of the microtip. The most important is microtip partial delamination and the next two are monomer mixture related as gas bubbles and compounds aging. In Figure 9, three SEM images of microtips illustrate the technological issues identified above. Interfacial delamination of a microtip from the optical fiber end face was observed in many experiments (Figure 9a). When delamination occurs, the VOC infiltrates free space between the microtip and optical fiber and condenses in this zone. This phenomenon distorts the measurement and reduces reflection. It was observed that partial delamination of the microtip can lead to a complete detachment of the microtip. If the gas bubbles appear in the monomer mixture, they result in large pores in the surface of the microtip (Figure 9b) which directly influence reflection properties of the microtip. The compound’s aging is manifested by the grain structure of the microtip (Figure 9c) which strongly scatters the light passing through the polymer structure and which, in turn, may reduce return losses. Based on the analysis of the test results and SEM photos, it turned out that microtips with a degraded surface structure also exhibit the required optical properties.

**Figure 10:**
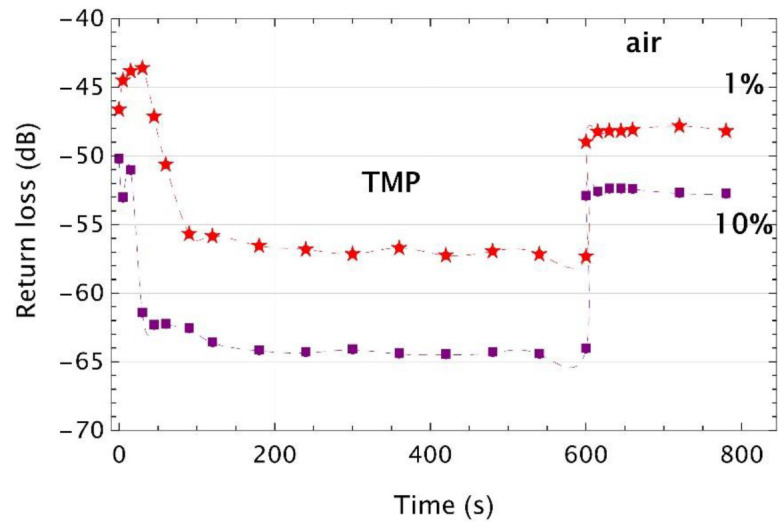
Time responses of the VIS microtips made with the monomer mixture enriched with TEOS cross-linking additive and with APTES treated optical fiber exposed to vapors of the TMP of a volume of 50 µL. Markers are experimental points, while the dashed lines are their cubic polynomial interpolations.

**Figure 11:**
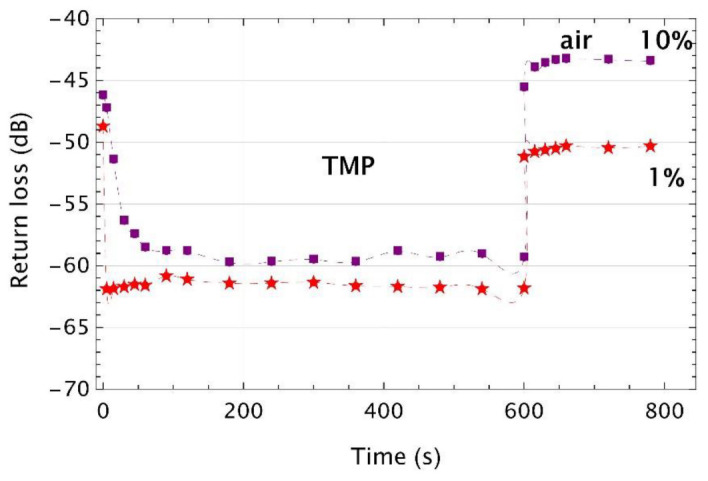
Time responses of the UV microtips made with the monomer mixture enriched with TEOS cross-linking additive and with APTES treated optical fiber exposed to vapors of the TMP of a volume of 100 µL. Markers are experimental points, while the dashed lines are their cubic polynomial interpolations.

In the next stage of the experiment, tetraethyl orthosilicate (TEOS) was added to the monomer mixture. This compound influences the polymer cross-linking process during polymerization. There were tested two concentrations of TEOS in the mixture, i.e., 1% and 10%. Moreover, the end face surface of the optical fiber was functionalized by using (3-aminopropyl)triethoxysilane (APTES) which improves the optical fiber surface ability to form a chemical bond with the polymer [42,43]. Application of APTES in the manufacturing process was a 2-min soaking of the optical fiber in its solution. Figure 10 and Figure 11 show the experimental results of VIS and UV microtips for which the monomer mixture was enriched with TEOS cross-linking additive, and the optical fiber was treated with APTES and exposed to TMP vapors. Experimental data were obtained for mixtures composed with 1% or 10% of TEOS (red- and purple-colored markers, respectively). Microtips were exposed to two TMP volumes of 50 μL (VIS microtips) and 100 μL (UV microtips). Measurement procedure was as follows. First, each transducer was measured in the air and then, it was inserted into the glass vial with the TMP and kept there for 600 s. In the last step, the microtip was put out from the vial and was measured for next 200 s.

Time responses in Figure 10 are consistent with previously presented results. Dynamic ranges of both tested VIS microtips were of around 10 dB and 12.5 dB of 1% and 10% of TEOS, respectively. The difference between the initial return losses of both tested microtips confirms that even with special care in the manufacturing process, this value may be lower than expected.

The dynamic ranges for UV microtips calculated based on data presented in Figure 11 were of around 10 dB between the first and second steps of the measurement procedure and were reduced to 6 dB for 1% of TEOS and 8 dB for 10% of TEOS between the second and third steps of this procedure.

Similarly to the previous results, initial values of return loss of the UV microtips-based sensors were not kept at the same level when the sensor was first inserted into the vial and next taken out from it. Thus, dynamic ranges of the first stage of the experiment were of around 11 dB for 1% of TEOS and 12 dB for 10% of TEOS. In the second part of the experiment, dynamic ranges were of around 6 dB for 1% of TEOS and 10 dB for 10%.

Comparing the above results, two conclusions can be drawn. First, the TEOS crosslinking additive does not significantly affect the operation of the sensor. Therefore, further studies were conducted without this compound. The second significant observation was that the change in environment causes a different response from the sensor. Every time when the optical fiber end with microtip changes environment from air to VOC and vice versa, the return loss has a different value. The signal level decreases when the transducer is measured in the presence of TMP vapors, but when it is placed back in neutral environment, the signal returns to its initial level. APTES application to the optical fiber has reduced delamination but has not eliminated it completely.

Liquid TMP applied to the microtip surface changes the refractive index, therefore, the value of the reflected signal changes, too. The RI of the liquid TMP is approximately of 1.3967 [41] and referring to previous studies [35,37], the change of the RI from 1.0 (air) to 1.4 caused the reflected signal value change of around 15 dB. Taking into consideration the results presented above, the dynamic range obtained for TMP vapors is lower than for the liquid material. However, the time response is bistabile on/off, and the sensor with such transducer has a threshold character.

In the next step of the experiment, it was decided to check the repeatability of the sensor operation by performing a measurement in which the transducer was inserted and removed from the vial several times, when it was filled with a selected of liquid material which easily transfers to the vapor phase in normal conditions. For further tests, 1,4-thioxane (THX), acetone, toluene, and ammonia aqueous with 25% of water were selected. The latter is an inorganic compound which was used to simulate another type of vapor, and which has the RI close to water.

Tests were carried out in a similar method as before. The microtip was inserted two times in a vial filled with the selected VOC. Experiment had two cycles, each consisting of two steps: measurement in the air and in the presence of VOC. Each experiment took 525 s and started in the air. Based on the analysis of SEM images, it was found that microtips manufacturing with the use of a UV LED gives a better effect since the element with a larger size and a smooth surface was obtained. Therefore, further research was limited to using only the UV source.

**Figure 12:**
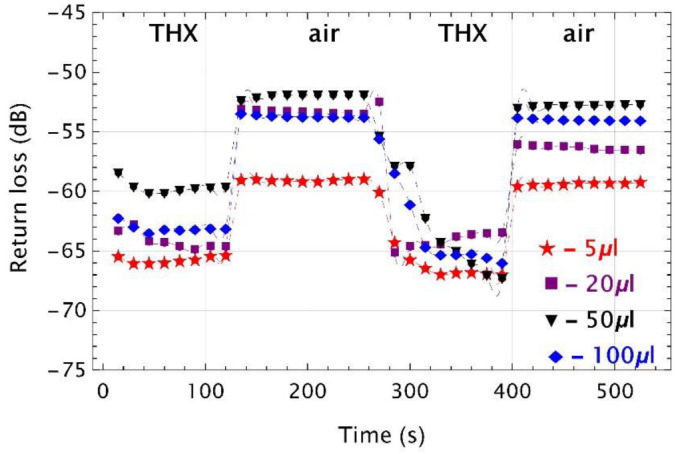
Time responses of UV microtips twice inserted into and removed from the vial filled with four different volumes of 1,4-thioxane (THX). Colored plot markers indicate experimental points, while the dashed lines are data interpolations.

Figure 12 shows the results of return loss measurements for UV microtips, for four THX concentrations (i.e., 5 μL, 20 μL, 50 μL, and 100 μL). THX belongs to the group of organosulfur compounds, for which the RI is of 1.5095 (*Merck* data sheet).

For this compound, as for TMP, the sensor has a bistabile on-off work. For a 5 μL THX, the dynamic range of return loss was of about 7 dB in both cycles. For 20 μL, this change was of around 11 dB in the first cycle and of 8 dB in the second one. Immersion of the microtip in a vial filled with 50 μL of THX resulted in dynamic ranges of 7 dB and 15 dB in the first and second cycles, respectively. While for 100 μL, these variations were of about 10 dB and 12 dB. It can be seen that for the selected VOC volumes, differences are not significant and depend largely on the shape and surface of the microtip. It is not possible to determine the vapor concentration level, and no correlation was found between signal level and VOC concentration.

**Figure 13:**
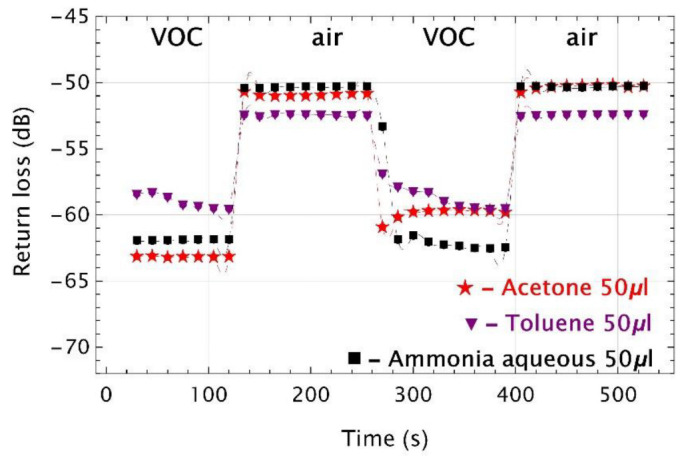
Time responses of UV microtips twice inserted into and removed from the vial filled with acetone (red), toluene (violet), and ammonia aqueous (black). Colored plot markers indicate experimental points, while the dashed lines are data interpolations. All VOCs are in a volume of 50 μL.

The next three tested compounds (i.e., acetone, toluene, and ammonia aqueous) are well known chemicals with the refractive indices of: 1.3588, 1.4941, and 1.3472 [41], respectively. Return loss measurement for acetone, toluene, and ammonia aqueous, all in concentrations of 50 μL, were presented in Figure 13.

The time responses for all tested compounds had a bisable on–off work as for previously tested VOCs. In the first cycle, the dynamic ranges were of around 13 dB, 11 dB, 7 dB, for acetone, toluene, and ammonia aqueous, respectively. While in the second cycle, these shifts were of 9 dB, 12 dB, and 7 dB.

Thus, the dynamic ranges of toluene and ammonia aqueous were at the same level and for acetone they differed. Moreover, toluene vapors needed more time to condense in both measurement cycles while for all of them return loss fluctuations in the second cycle are visible. However, again, dynamic ranges do not correlate with RIs values of the tested material.

Since the polymer material is soluble in acetone [44], it was decided to check how the local deposition of condensed acetone influences the disruption of the microtip surface. Microtip was left for 2 h in a vial of acetone vapor and its surface structure was checked based on SEM images. No changes in the surface morphology and shape were found. It is likely that the thin layer of acetone deposited on the surface is not sufficient to etch the polymer to make any cavities in the microtip.

## 5. Discussion

The main goal of the paper was to evaluate feasibility of the polymer microtip as a transducer of the VOC sensor. To achieve this goal, the polymer microtips from mixtures cured with UV and VIS light were produced, technological parameters of fabrication process were corrected, and five selected volatile compounds were tested.

Manufactured microtips were first exposed to TMP with different concentrations. Based on these tests, it was found that the proposed sensor works as the threshold sensor, showing only the presence of the VOC but without possibility to determine its concentration. After some corrections to the technological parameters described later in this paragraph, the testes were continued. In the next stage of the experiments, the periodic exposition of the sensor transducers to the selected VOCs were performed. Then, THX was selected due to its RI which is higher than TMP. This was conducted based on previously reported studies which had shown that the tested sensor can be used as a RI sensor [37]. THX was measured with four different concentrations. There were visible changes of the measured backscatter signal level when the microtip was exposed to the THX or to the air, but correlation between volumes of the selected VOC and return loss was negligible. Further, there were applied two well-known VOCs (i.e., acetone and toluene) and one inorganic volatile compound (i.e., 25% ammonia solution). For the first time, the measured time responses for all of the mentioned materials were also comparable with the previously obtained results for TMP and THX. Therefore, considering the above, the tested sensor can only be considered a threshold sensor.

In the authors’ opinion, the main reason for such a working principle of the sensor is the condensation process of volatile compound on the microtip surface. Phase transition from gas to liquid of the tested VOC or even inorganic ammonia aqueous on the microtip changes the RI on the interface of these materials. The effect of this is the change of the measured return loss level. However, the condensed material can be nonuniformly distributed on the microtip since it forms liquid drops with small sizes and thicknesses [38]. Since the RIs difference between liquid and polymer is lower than between gas and polymer, a decrease in the back-reflected signal is observed. The number of vapors of the tested volatile material in the vial depends on its physical properties and the experiment conditions. A selected volume of the tested material was introduced into the vial and allowed to stand for approximately 1 h to reach thermodynamic equilibrium between the liquid and gas phases before the measurement starting. The experimental results have shown that the change in the back-reflected signal caused by the material condensation is comparable for all of the tested samples. Therefore, it was not possible to determine which compound was tested or what was its concentration. Finally, these measurements allow one to categorize this sensor as a condensed material threshold sensor.

During these experiments, the problems of delamination and abnormal surface morphology of the microtips were encountered. These problems have led to an extended evaluation of the technological process parameters. TEOS crosslinking additive was used for the monomer mixture and APTES was used to functionalize the glass surface of the optical fiber. TEOS applied in two different concentrations did not confirm a positive influence on the sensor sensitivity.

However, functionalization of the optical fiber end face by using APTES improved the ability to form a chemical bond between the optical fiber surface and the polymer and reduced delamination, but did not eliminate it completely.

Taking into account all of the presented experimental results, the proposed sensor can be used in areas of monitoring accumulated hazardous liquid substances that easily transition to the vapor phase. It will then act as an early warning system for the condensation process of a monitored hazardous chemical agent (for example, to control the possible leakage from a pipeline containing volatile compound).

## Data Availability

Data available on request from the corresponding author.
